# miR-214-3p Is Commonly Downregulated by EWS-FLI1 and by CD99 and Its Restoration Limits Ewing Sarcoma Aggressiveness

**DOI:** 10.3390/cancers14071762

**Published:** 2022-03-30

**Authors:** Alessandra De Feo, Laura Pazzaglia, Lisa Ciuffarin, Fabio Mangiagli, Michela Pasello, Elisa Simonetti, Evelin Pellegrini, Cristina Ferrari, Giuseppe Bianchi, Benedetta Spazzoli, Katia Scotlandi

**Affiliations:** 1 SSD Laboratory of Experimental Oncology, IRCCS Istituto Ortopedico Rizzoli, Via di Barbiano 1/10, 40136 Bologna, Italy; laura.pazzaglia@ior.it (L.P.); lisaciuff22@hotmail.it (L.C.); fabio.mangiagli@hotmail.it (F.M.); michela.pasello@ior.it (M.P.); elisa.simonetti@ior.it (E.S.); evelin.pellegrini@ior.it (E.P.); cristina.ferrari@ior.it (C.F.); 2 IRCCS Istituto Ortopedico Rizzoli, Third Orthopaedic Clinic and Traumatology, 40136 Bologna, Italy; giuseppe.bianchi@ior.it (G.B.); benedetta.spazzoli@ior.it (B.S.)

**Keywords:** Ewing sarcoma, miR-214-3p, HMGA1, EWS-FLI1, CD99, migration

## Abstract

**Simple Summary:**

Ewing’s sarcoma (EWS), the second most frequent primary tumor of bone in the pediatric population, is a very aggressive, undifferentiated mesenchymal malignancy with a high tendency to develop lung and/or bone metastasis. The prognosis of patients with metastasis remains dismal, and new strategies are needed to control the dissemination of EWS cells. EWS is driven by alterations induced by the EWS-FLI1 chimera which acts as an aberrant transcriptional factor that induces the complete reprograming of the gene expression. EWS cells are also characterized by high expression of CD99, a cell surface molecule that interacts with EWS-FLI1 to sustain EWS malignancy. This study shows that miR-214-3p is a common mediator of EWS-FLI1 and CD99, and we report that miR-214-3p acts as on oncosuppressor in EWS. MiR-214-3p is constitutively repressed in cell lines and clinical samples but is re-expressed after the silencing of EWS-FLI1 and/or CD99. The restoration of miR-214-3p limits EWS cell growth and migration and represses the expression of its target HMGA1, supporting the potential role of this miRNA as a marker of tumor aggressiveness.

**Abstract:**

Ewing’s sarcoma (EWS), an aggressive pediatric bone and soft-tissue sarcoma, has a very stable genome with very few genetic alterations. Unlike in most cancers, the progression of EWS appears to depend on epigenetic alterations. EWS–FLI1 and CD99, the two hallmarks of EWS, are reported to severely impact the malignancy of EWS cells, at least partly by regulating the expression of several types of non-coding RNAs. Here, we identify miR-214-3p as a common mediator of either EWS-FLI1 or CD99 by in silico analysis. MiR-214-3p expression was lower in EWS cells and in clinical samples than in bone marrow mesenchymal stem cells, and this miRNA was barely expressed in metastatic lesions. Silencing of EWS-FLI1 or CD99 restored the expression of miR-214-3p, leading to a reduced cell growth and migration. Mechanistically, miR-214-3p restoration inhibits the expression of the high-mobility group AT-hook 1 (HMGA1) protein, a validated target of miR-214-3p and a major regulator of the transcriptional machinery. The decrease in HMGA1 expression reduced the growth and the migration of EWS cells. Taken together, our results support that the miR-214-3p is constitutively repressed by both EWS-FLI1 and CD99 because it acts as an oncosuppressor limiting the dissemination of EWS cells.

## 1. Introduction

Ewing’s sarcoma (EWS), the second most common bone tumor in childhood and adolescence, is a highly aggressive and poorly differentiated neoplasm with a high tendency to metastasize. Histologically, EWS comprises undifferentiated, small round cells expressing high levels of CD99, a cell surface molecule that regulates crucial biological processes, including cell adhesion, migration, and metastases [1]. Genetically, EWS is characterized by a recurrent balanced chromosomal translocation, most frequently the *t*(11;22) (q12;q24) which results in the fusion of the *EWSR1* gene with the *ETS* family gene *FLI1*. This fusion transcript has been widely proven to be the genetic hallmark of EWS and its oncogenic driver [2]. Additional genetic alterations are rare [3,4,5].

Patients with localized EWS have a survival rate of 70% because of intensified cytotoxic drug regimens [6], but this intense treatment is frequently associated with side effects that severely impact the quality of life of the survivors. Additionally, patients with localized disease and a favorable prognosis face the enduring threat of late relapses due to metastasis formation. Patients who fail to respond to first-line treatments or who already have metastases at diagnosis have a dismal prognosis, with an overall survival rate of less than 40%. The presence of metastases is the most powerful adverse prognostic factor in EWS and novel treatments devoted explicitly to preventing their formation or eradication are needed. However, the underlying mechanisms responsible for the dissemination and distal growth of EWS cells remain poorly understood.

Extensive research and a rapidly growing literature base are available on the role of cellular plasticity rather than gene mutations in regulating the metastatic processes in EWS [2,7]. Comprehensive genomic and epigenomic profiling has revealed that EWS–FLI1 drives widespread epigenetic reprogramming by inducing de novo EWS-specific enhancers and repressing enhancers that are active in many cell types [8,9,10]. This evidence, together with evidence concerning the few genetic alterations detected in EWS [3,4,5], indicates that epigenetic factors likely play a critical role in EWS initiation and progression.

MicroRNAs (miRNAs), a class of evolutionarily conserved small non-coding single-stranded RNA molecules that play a pivotal role in post-transcriptional gene silencing are part of the epigenetic machinery, and their role in the pathogenesis and progression of EWS has been demonstrated [11]. Many non-coding RNAs are regulated by EWS–FLI1 and are key players in EWS tumorigenesis [12], potentially providing novel biomarkers and therapeutic approaches. In addition to EWS-FLI1, CD99 the other hallmark of EWS, was found to modulate the expression of miRNAs [13,14]. Knockdown of either EWS-FLI1 or CD99 by short interfering RNA (siRNA) induces the growth arrest, apoptosis, and differentiation of EWS cells and significantly inhibits tumorigenesis and metastasis [15,16]. EWS-FLI1 participates in maintaining the high expression of CD99 not only through transcriptional regulation of CD99 but also through indirect mechanisms, such as the repression of miR-30a-5p, which targets the 3′ untranslated region of CD99 [17], supporting evidence that crosstalk between these two molecules is required for the malignancy of this tumor. In this study, we focused on identifying miRNAs that are commonly regulated by EWS-FLI and CD99. We exploited publicly available datasets that reported miRNA signatures associated with EWS-FLI1 [18,19] or CD99 silencing [13] and identified miR-214-3p as a shared repressed miRNA. This miRNA is associated with major biological processes, such as cell proliferation, stemness regulation, invasiveness, and metastasis, and resistance to chemotherapy in several types of cancer [20,21,22,23,24]. Mechanistically, miR-214-3p behaves as a hub coordinating fundamental signaling networks [25], including those involving phosphatase and tensin homolog (PTEN) [26], beta-catenin [27], and Yes-associated protein/transcriptional co-activator with PDZ binding motif (YAP/TAZ) signaling [28]. In the musculoskeletal system, its altered expression is associated with some pathological conditions of the bone, such as osteoarthritis, bone remodeling and osteoporosis [29,30]. In bone tumors, miR-214 plays dual roles: it acts as an oncomiR in osteosarcoma [31,32] and osteolytic bone metastasis of breast cancer [33] and as an oncosuppressor in myeloma cells [34]. Our study reports that miR-214-3p acts as on oncosuppressor in EWS: it is constitutively repressed in cell lines and clinical samples but is re-expressed after the silencing of EWS-FLI1 and/or CD99. The restoration of miR-214-3p limits EWS cell growth and migration and represses the expression of high mobility group AT-hook 1 (HMGA1), a small non-histone protein that modifies the access of regulatory factors to the DNA, contributing to the overall regulation of gene expression [35].

## 2. Material and Methods

### 2.1. Cell Lines and Primary Cultures

EWS human cell line TC-71 cells were kindly provided by T.J. Triche (Children’s Hospital, Los Angeles, CA, USA) and SK-N-MC (ATCC Cat# CRL-2270), SK-ES-1 (CLS Cat# 300435/p738_SK-ES-1) and RD-ES (CLS Cat# 300410/p763_RD-ES) were purchased from the American Type Culture Collection (ATCC). IOR-CAR, IOR-BRZ and LAP-35 cells were previously established in our laboratory [36], and A673 cells (CLS Cat# 300454/p491_A-673) were provided by H. Kovar (St. Anna Children’s Cancer Research Institute, Vienna). PDX-EW#2-C cells and PDX-EW#5-C cells were established from the corresponding EWS Patient-Derived-Xenografts (PDXs) after the first passage in animals as previously described [37]. Human mesenchymal stem-cell bone-marrow derivatives were kindly provided by E. Lucarelli (Istituto Ortopedico Rizzoli, Bologna, Italy) and cultured as previously described [38,39]. EWS cells were cultured in Iscove’s modified Dulbecco’s medium (IMDM; ECB2072L, EuroClone, Milan, Italy) supplemented with 10% fetal bovine serum (FBS) (ECS0180L, EuroClone) 100 U/mL penicillin and 100 mg/mL streptomycin (Merck) and incubated at 37 °C in a humidified atmosphere containing 5% CO_2_. All the cell lines were assessed for mycoplasma contamination (MycoAlert Mycoplasma Detection Kit, Lonza, Basel, Switzerland) before starting experiments and were authenticated by short tandem repeat (STR) polymerase chain reaction (PCR) analysis by using the PowerPlex ESX Fast System kit (DC1710, Promega, Madison, WI, USA) (last control December 2017 and July 2018). The ethics committee of the Rizzoli Institute approved the establishment of PDX models (0009323/2016).

### 2.2. Anti-CD99 Antibody Treatment

The hybridoma used to produce the murine anti-CD99 mAb 0662 was kindly provided by Alain Bernard (Unite INSERM 343, Hospital de l’Archet, Nice, France). A total of 200,000 cells were seeded in 60-mm diameter petri dishes. Forty-eight hours later, they were treated with mAb 0662 (3 μg/mL) for 3 to 6 h before harvesting and processing for quantitative PCR (qPCR) and in vitro experiments, as described in the following sections.

### 2.3. Stable and Transient Transfection

Stable CD99 silencing was achieved in TC-71 cells as previously described [14,40]. Stable inducible CD99 silencing was achieved through sequential transfection of A673 cells with the plasmid pcDNA/6TR (Thermo Fisher Scientific, Waltham, MA, USA), encoding the reverse tetracycline (TET)-responsive transcriptional activator and the plasmid pTER vector (kindly provided by K. Laud-Duval, Institut Curie, Paris, France), and engineered in our laboratory to express CD99 shRNA as previously described [41]. The A673/TR/shEF cell line, generated from A673 EWS cell line after stable transfection with doxycyclin-inducible shRNA targeting EWS-FLI1 [42] was cultured in complete medium supplemented with 20 μg/mL of blasticidin (Invitrogen, Grand Island, NY, USA) and 50 μg/mL of zeocin (Sigma-Aldrich, St Louis, MO, USA).

Stable clones SK-N-MC #12 and SK-N-MC #34, kindly provided by H. Kovar (St. Anna Children’s Cancer Research Institute, Vienna, Austria), were obtained using the miR-214 plasmid created by PCR amplification using human genomic DNA as a template. The primers are the following: 5′-CACCTTTCTCCCTTTCCCCTTACTTACTCTCC-3′(sense) and 5′-TGCCTTTCCCCAGTGCCTCTTTCTC-3′ (antisense). The PCR products (392bp containing pri-miRNA) were cloned into pcDNA3.1/V5-His-Topo-expression vector (Invitrogen, Waltham, MA, USA) and confirmed by DNA sequencing. The expression of miRNA was carried out by transfection of the plasmid into cells by using Lipofectamine 2000 (Thermofisher Scientific, Waltham, MA, USA).

TC-71, IOR-CAR, and PDX-EW#2-C cells were transfected with pre-miR-214-3p mimic, and PDX-EW#5-C cells with antagomiR-214-3p or with nonspecific control miRNAs (SCR) (30 nM) (assays #AM17100 and #AM171000; Ambion, Austin, TX, USA) by using a TransIT-X2 Dynamic Delivery System (Mir6000, Mirus, Madison, WI, USA) 24 h after cell seeding. The expression level of miR-214-3p was determined by qPCR up to 48 h after transfection by using the ΔCT relative method [43].

Transient silencing of HMGA1 was performed by using the short interfering RNA (siRNA) SMART POOL siGENOME_siRNA (M-004597-02-0020 GE Healthcare Dharmacon, Lafayette, CO, USA). SiGENOME_non-targeting siRNA was used as a control (D-001206-13-05, GE Healthcare Dharmacon). SiRNAs (20 nmol/L) were transfected into PDX-EWS #5-C by using TransIT-X2 according to the manufacturer’s protocol. Cells were harvested for qRT-PCR analysis 72 h after transfection.

### 2.4. Clinical Samples

miR-214-3p expression was evaluated by qPCR in 23 frozen tissue specimens derived from localized primary tumors and in 21 metachronous metastases (13 lung, 6 bone, 1 lymph node, and 1 spread) from a cohort of patients with confirmed diagnosis of EWS treated at the Istituto Ortopedico Rizzoli (Bologna, Italy). The ethics committee of the Istituto Rizzoli approved the study (0019012/2016; 505/2019), and informed consent was obtained. The study was conducted in accordance with the Declaration of Helsinki ethical guidelines.

### 2.5. RNA Extraction and Quantitative PCR

RNA from cell lines and tissues was extracted by using the TRIzol reagent following the manufacturer’s instructions (Life Technologies, Grand Island, NY, USA) and nucleic acid quality and quantity were assessed by using a NanoDrop spectrophotometer (NanoDrop Technologies LLC, Wilmington, DE, USA). The total RNA from each sample was reverse-transcribed into cDNA by using the High-Capacity cDNA Reverse Transcription Kit (Life Technologies, Carlsbad, CA, USA) according to the manufacturer’s protocols. qRT-PCR was performed by using a ViiA7 system (Life Technologies) and TaqMan PCR Master Mix (Life Technologies). Predesigned TaqMan probes (Life Technologies) were used for miR-214-3p (assay ID: 002306) and for HMGA1 (assay ID: Hs00431242_m1).

Relative quantification was performed by using the ΔCT method, the expression levels of the target genes were normalized to those of the housekeeping gene glycer-aldehyde-3-phospate dehydrogenase (*GAPDH*) (assay ID: Hs99999905_m1), or *RNU6B* (assay ID: 001093) (Life Technologies).

### 2.6. Cell Growth Assays

A total of 20,000 cells/cm^2^ were seeded to determine the cell viability by using the trypan blue vital cell dye (Sigma-Aldrich, St Louis, MO, USA) over 7 days.

Anchorage-independent growth was determined in 0.33% agarose (SeaPlaque, FMC BioProducts, Rockland, ME, USA) with a 0.5% agarose underlay. Cell suspensions (3300 cells) were plated in 60-mm diameter petri dishes in a semisolid medium IMDM 10% FBS. Dishes were incubated at 37 °C in a humidified atmosphere containing 5% CO_2_. Colonies were counted after 7 days.

### 2.7. Migration Assay

Migration ability of the EWS cell lines was assessed by using transwell chambers (Costar, Cambridge, MA, USA) polycarbonate filters containing 8 μm pore size. A total of 100,000 cells of TC71, SK-N-MC, and PDX-EW#2-C were seeded in the IMDM plus 10% FBS. IOR-CAR and PDX-EW#5-C cells with a low capability to migrate were seeded in 1% FBS in the upper compartment, and IMDM 20% FBS was placed in the lower compartment (gradient). After 16 h of incubation at 37 °C in a humidified atmosphere, the migrated cells were fixed with methanol, stained with Giemsa dye and counted at 10× magnification.

### 2.8. Western Blotting

Harvested cells were rinsed in 1X phosphate-buffered saline (PBS) and lysed in phospho-protein extraction buffer supplemented with protease-phosphatase cocktail inhibitor (Sigma, St Louis, MO, USA). Western blotting was performed according to standard procedures. Equivalent amounts of lysates (40 µg) were run on sodium dodecyl sulfate (SDS) gels under denaturing conditions and blotted onto nitrocellulose membranes. The membranes were incubated overnight with the following primary antibodies: anti-HMGA1 (1:5000 cat:129153, Abcam, Cambridge, MA, USA), anti-actin (1:10,000 cat:MAB1501, Millipore, Burlington, MA, USA), anti –IGF-1R (1:1000 cat: SC-390130, Santa Cruz Biotechnology, Dallas TX USA), anti-DDR1 (1:1000 cat: SC-532 Santa Cruz Biotechnology, Dallas TX USA), anti-ERK1/2 (1:5000 cat: #9102 Cell Signaling, Boston, MA, USA), and anti- AKT (1:3000 cat: #9272, Cell Signaling, Boston, MA, USA).

Anti-rabbit (NA934) and anti-mouse (NA931, GE Healthcare, Little Chalfont, UK) antibodies conjugated to horseradish peroxidase were used as secondary antibodies. The signal was visualized by using enhanced chemiluminescence (ECL) reagents (LiteAblot PLUS, Euroclone, Milan, Italy or ThermoScientific Super Signal west PicoPlus) and quantified by densitometric analysis by using a GS-800 imaging densitometer and Quantity One software (Bio-Rad, Hercules, CA, USA).

### 2.9. Statistical Analyses

All statistical analyses were performed by using Prism version 7.0 (GraphPad Software, La Jolla, CA, USA). Differences among means were evaluated by one-way analysis of variance (ANOVA) whereas two-tailed Student’s *t*-tests were used for comparisons between two groups. The data was considered statistically significant at *p* < 0.05.

### 2.10. Software Analysis

Venny 2.1.0 software (https://bioinfogp.cnb.csic.es/tools/venny) was used to identify overlapping miRNAs in three public miRNA databases [13,18,19].

## 3. Results

### 3.1. miR-214-3p Is Commonly Repressed by EWS-FLI1 and CD99 and Restoration of Its Expression Stalls Tumor Cell Growth and Cell Migration

**Figure 1:**
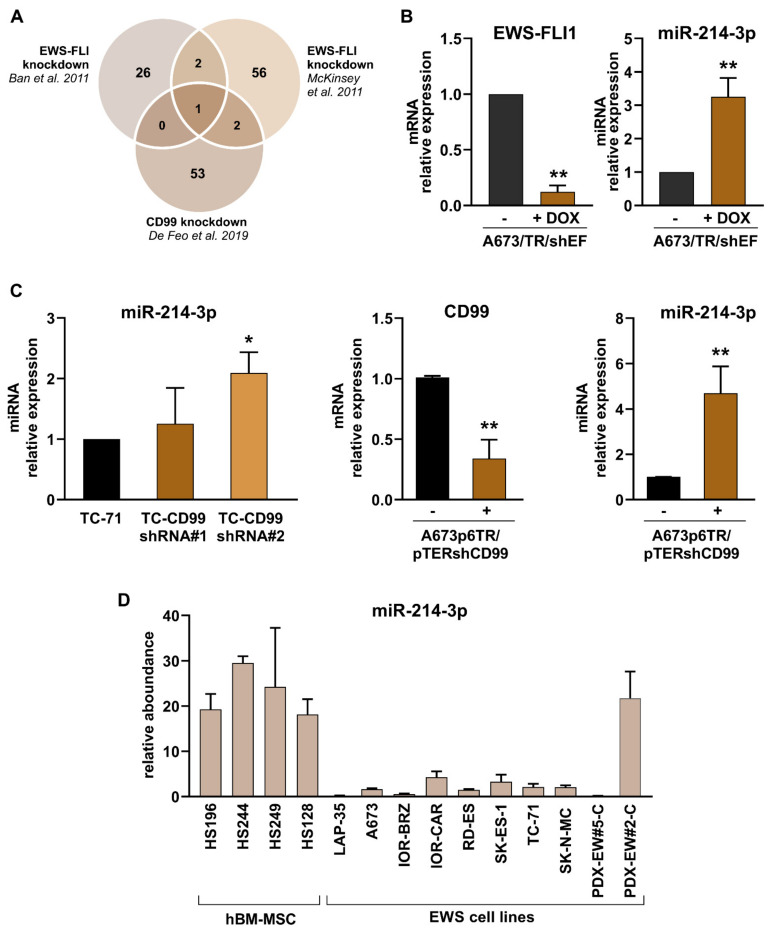
Effect of *EWS-FLI1* and *CD99* silencing on miR-214-3p expression. (**A**) The Venn diagram shows that miR-214-3p was the only common miRNA for EWS-FLI and CD99 from three miRNA data-bases. (**B**) Evaluation of miR-214-3p and *EWS-FL1* expression by qRT-PCR in EWS-FLI1-silenced cells, A673/TR/shEF. (**C**) Evaluation of miR-214-3p and in CD99-silenced TC-CD99-shRNA cells (stable model; C, left); *CD99* and miR-214-3p expression inA673p6TR/pTERshCD99 cells (inducible model; C, right). (**D**) MiR-214-3p expression analysis by qRT-PCR in a panel of human patient-derived cell lines (LAP-35, A673, IOR-BRZ, IOR-CAR, RD-ES, SK-ES-1, TC-71, SK-N-MC), PDX-derived cell lines (PDX-EW#5-C, PDX-EW#2-C) and human bone-marrow-derived mesenchymal cells (HS196, HS244, HS249, HS128). The data are shown as the mean ± SE of three independent biological experiments (* *p* < 0.05, ** *p* < 0.01 Student’s *t* test).

We exploited publicly available data from EWS cells with EWS-FLI1 or CD99 silencing to perform an in silico analysis as a first step in identifying miRNAs that are commonly regulated by the two hallmarks of EWS. A Venn diagram (Figure 1A and Supplementary Table S1) showed that only one miRNA was shared among the three available miRNA databases [13,18,19]: the miR-214-3p. To confirm that the miR-214-3p expression depends on EWS-FLI1 and on CD99, we silenced EWS-FLI1 (Figure 1B) or CD99 (Figure 1C) in EWS cell lines and observed increased expression of miR-214-3p (Figure 1B,C) following both the conditions. This finding indicates that miR-214-3p has a possible oncosuppressive role in EWS. Accordingly, the expression of the miR-214-3p was highly expressed in human bone marrow-derived mesenchymal stem cells (hBM-MSCs), which are considered the putative cells of origin of EWS [44], but barely expressed in EWS patient-derived cell lines, with the exception of one (Figure 1D). The panel of cell lines includes novel EWS cell lines established from patient-derived xenografts, which faithfully model patient tumors [37,45] in experimental studies.

**Figure 2:**
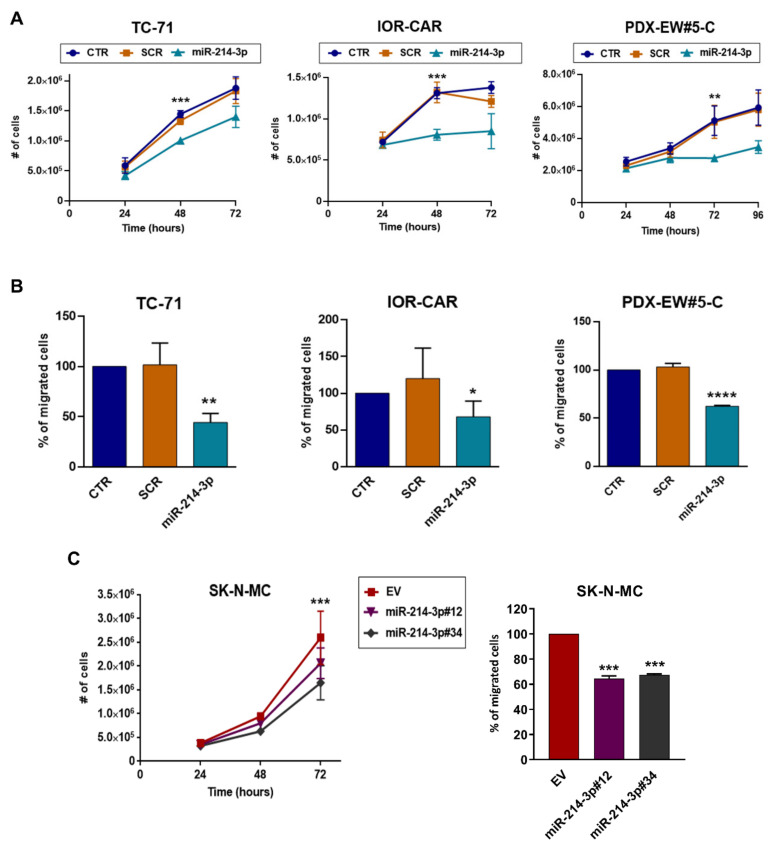
MiR-214-3p acts as an oncosuppressor in EWS suppressing tumor malignancy. Transient transfection with miR-214-3p mimic (30 nM) reduced cell growth (**A**) and migration (**B**) of TC-71, IOR-CAR and PDX-EW#5-C cells (CTR, non-transfected cells; SCR, non-specific control miRNAs). SK-N-MC cells stably transfected with the empty vector (EV, control) or with miR-214-3p (overexpressing variants #12 and #34) were compared for the analysis of in vitro cell growth ((**C**), left) and cell migration ((**C**), right). The data are shown as the means ± SE of three independent biological experiments performed in duplicate (* *p* < 0.05, ** *p* < 0.01, *** *p* < 0.001, **** *p* < 0.0001, one-way ANOVA).

**Figure 3:**
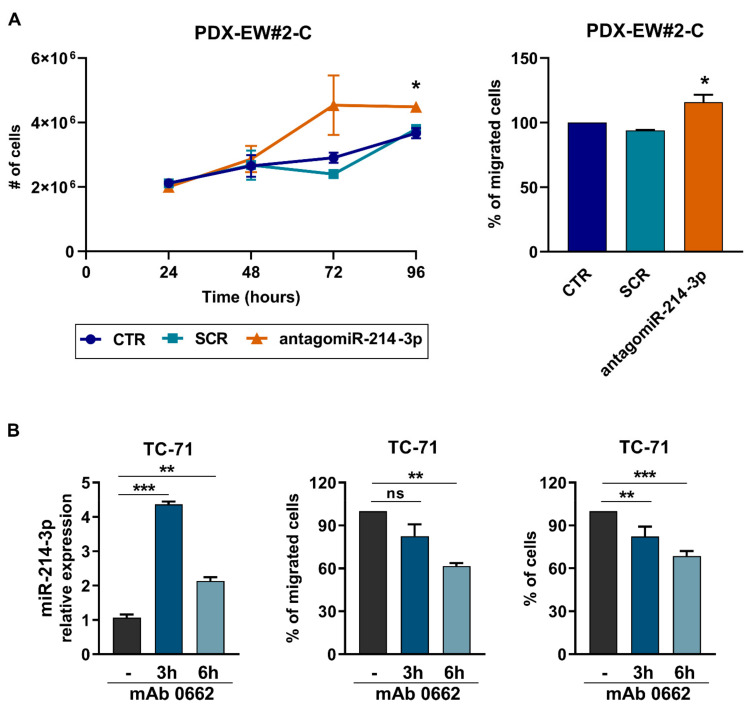
Treatment of EWS cell lines with antagomiR-214-3p and anti-CD99 mAb 0662. (**A**) Transient transfection of antagomiR-214-3p (30 nM) increased cell growth (left) and cell migration (right) on the PDX-EWS#2-C cell line. The data are shown as the mean ± SE of three independent biological experiments performed in duplicate (* *p* < 0.05, one-way ANOVA); (CTR, nontransfected cells; SCR, nonspecific control antagomiR). (**B**) Evaluation of miR-214-3p expression, cell migration and tumor growth in TC-71 cells after treatment with the anti-CD99 antibody (mAb 0662 3 µg/mL). Data are shown as 2^−ΔΔCt^ using TC-71 cells for normalization, and *RNU6B* as endogenous control for miR-214-3p expression. The data are shown as the mean ± SE of three independent biological experiments performed in duplicate (ns: not significant, * *p* < 0.05, ** *p* < 0.01, *** *p* < 0.001 one-way ANOVA).

To assess the role of miR-214-3p, we transiently induced its expression in TC-71, IOR-CAR, and PDX-EWS#5-C in EWS cells or stably forced its expression in SK-N-MC cells. In all cases, miR-214-3p overexpression resulted in stalled growth under standard-2D (Figure 2A,C left) or anchorage-independent 3D growth conditions (Supplementary Figure S2A,B) and decreased migration (Figure 2B,C right). Conversely, transfection of the anti-miR-214-3p into the PDX-EWS#2-C EWS cell line, which showed the highest expression of miR-214-3p, resulted in a modest but significant increase in cell growth and migration (Figure 3A,B). In addition, to reinforce the relationship between CD99, miR-214-3p expression and EWS malignancy, we induced triggering of CD99 with the anti-CD99 antibody (mAb 0662). We have already demonstrated that engagement of CD99 with antibodies led to deprivation of CD99 from the cell membrane [41,46]. In keeping with data obtained by gene modifications, we observed an upregulation of miR-214-3p followed by a reduction in cell migration and growth (Figure 3B).

**Figure 4:**
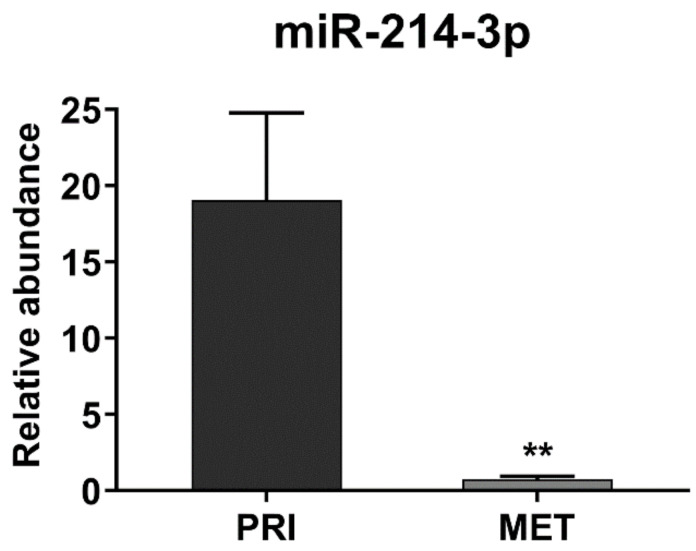
MiR-214-3p expression in clinical specimens. MiR-214-3p expression evaluated in EWS clinical samples by qRT-PCR. The analysis was performed by comparing primitive tumors (PRI, *n* = 23) and metastases (MET, *n* = 21). The data are shown as 2^−Δ^*Ct* using *RNU6B* as endogenous control (** *p* < 0.01, Student’s *t*-test).

Finally, to verify the clinical relevance of these findings, we compared the expression of miR-214-3p in samples derived from primary localized tumors (*n* = 23) or metastatic lesions (*n* = 21). The expression of miR-214-3p was significantly lower in metastasis than in primary tumors and was absent in some cases (Figure 4), confirming the inverse relationship between miR-214-3p expression and EWS aggressiveness in a clinical setting.

### 3.2. miR-214-3p Affects HMGA1 Expression

**Figure 5:**
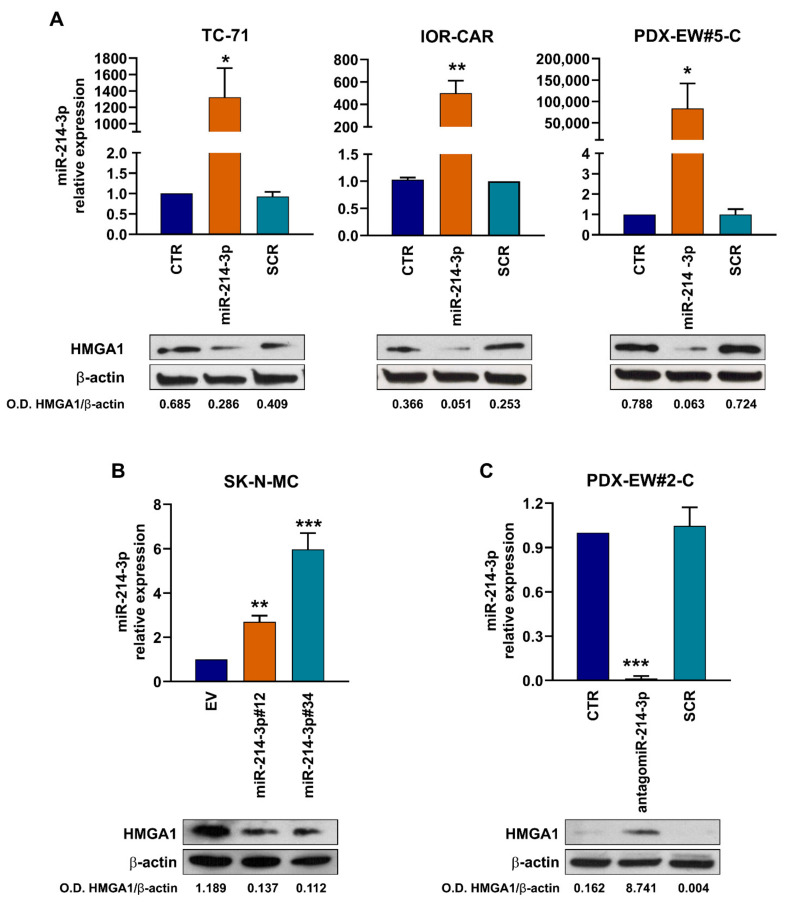
HMGA1 protein expression after either upregulation or inhibition of miR-214-3p. MiR-214-3p expression was analyzed by qRT-PCR and HMGA1 protein expression was analyzed by Western blotting: in TC-71, IOR-CAR and PDX-EW#5-C cells after miR-214-3p mimic treatment ((**A**) CTR, non-transfected cells; SCR, non-specific control miRNAs); in SK-N-MC empty vector (EV) and in the miR-214-3p-overexpressing variants (#12, and #34 (**B**)) and in PDX-EW#2-C cells (**C**). For qRT-PCR analysis, data are shown as 2^−ΔΔ^*Ct* using parental cell lines as calibrator and *RNU6B* as endogenous control. Mean *±* SE of three independent experiments is shown (* *p* < 0.05, ** *p* < 0.01, *** *p* < 0.001, one-way ANOVA). For HMGA1 protein expression analysis, β-actin was used as the loading control. O.D. HMGA1/β-actin represents the ratio of the volume-adjusted optical density of the HMGA1 signal to that of the actin signal.

**Figure 6:**
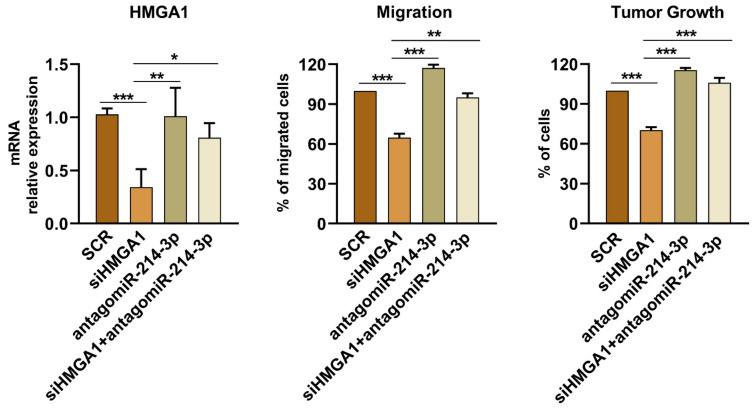
Effects of miR-214-3p and *HMGA1* silencing on cell migration and cell growth. Evaluation of *HMGA1* expression levels (**left**) and cell migration (**middle**) and tumor growth (**right**) on PDX-EWS#2-C after *HMGA1* silencing, antagomiR-214-3p, and combined treatment. The data are shown as 2^−ΔΔC*t*^ using SCR cells for normalization, with *GAPDH* as an endogenous control for *HMGA1* expression. The data are shown as the means ± SE of three independent biological experiments performed in duplicate (* *p* < 0.05, ** *p* < 0.01, *** *p* < 0.001 one-way ANOVA). SCR, non-specific control antagomiR plus scrambled control siRNA.

In cells overexpressing miR-214-3p, we analyzed the protein expression of some validated targets of miR-214-3p, such as DDR1, IGF-1R, AKT, and ERK signaling (Supplementary Figure S1) but we could not identify differences in EWS cells. Thus, we used publicly available bioinformatic target prediction tools (TargetScan 7.1 and DIANA-MicroT) and literature data to identify other candidate targets of miR-214-3p that may be responsible for the variations in cell growth and migration observed in EWS cells following the modulation of this miRNA. We focused our attention on the HMGA1 gene, a validated direct target of miR-214-3p in human cervical, colorectal, and endometrial cancer cell lines [47,48]. We evaluated the expression levels of HMGA1 after miR-214-3p up-/downregulation or after triggering of CD99 with antibody. As expected, increased expression of miR-214-3p in TC-71, IOR-CAR, and PDX-EW#5-C cells, or the SK-N-MC stable clones reduced HMGA1 expression (Figure 5A,B). Expression of HMGA1 was also reduced after engagement of CD99 with the anti-CD99 antibody (Supplementary Figure S3), demonstrating an inverse correlation with expression of miR-214-3p (Figure 3B). Conversely, when endogenous miR-214-3p was inhibited by using an antagomiR in PDX-EW#2-C cells, HMGA1 protein levels were increased (Figure 5C). In PDX-EW#2-C cells we also demonstrated that the silencing of HMGA1 (Figure 6, left) led to a significant reduction in cell migration and growth (Figure 6, middle and right). The inhibition of miR-214-3p by antagomiR restored the relative expression of HMGA1 (Figure 6 left) and cell growth and migration were re-established (Figure 6 middle and right). When PDX-EW#2-C cells were simultaneously exposed to antagomiR-214-3p and siHMGA1, effects on HMGA1 expression were found comparable to those obtained by using antagomiR only.

## 4. Discussion

EWS-FLI1 and CD99 are major triggers of cell growth, differentiation and migration of EWS cells, and fluctuation in their expression levels strongly affect the abilities of these cells to disseminate and form metastases [40,49,50].

Importantly, EWS-FLI1 expression changed over time in a fully reversible process to regulate the propensity of cells to proliferate and/or to migrate, invade and metastasize [49]. Several approaches have revealed a strong influence of EWS-FLI1 on the expression of proteins involved in cell cytoskeleton structure and cell adhesion and these modifications are associated with major phenotypic changes in tumor cells. Similarly, the modulation of CD99 affects cell growth, differentiation, migration, and metastasis by regulating intracellular signaling pathways and nuclear effector signals resulting from changes in the cell–cell or cell–matrix interactions [40,41]. Whereas in many other tumors metastatic spread is likely driven by accumulation of genetic alterations that provide cancer cells with the functional ability to disseminate and grow in distant sites, the metastatic process of EWS cells seems to be linked to cellular plasticity. Different groups have described the paucity of secondary genetic alterations [3,4,5]. Even if *STAG2* and *TP53* mutations are associated with a more aggressive disease, their low frequency cannot account for the high rate of metastasis, further reinforcing the idea that epigenetic regulation of gene expression plays a major role in in EWS tumor progression. Here, we focused on the miRNA expression variations observed to be commonly associated with both EWS-FLI1 and CD99. The comparison of the publicly available miRNA datasets associated with EWS-FLI1 deprivation and CD99 silencing identified miR-214-3p as the only common miRNA. This miRNA plays a dualistic role depending on the cellular context. In pancreatic cancer [51], stomach cancer [52], lung carcinoma [53], and in osteosarcoma [54] miR-214-3p generally is over-expressed. However in other tumors such as glioma [55], myeloma [56], and cervical cancer [21], its expression is generally low. Ban et al. showed decreased expression levels of this miRNA in five EWS samples compared with those in the mesenchymal progenitor cells of six healthy individuals [18]. In this study, we extended these finding by demonstrating a significant reduction in the expression of miR-214-3p in metastatic lesions compared with that in primary tumors. This result is consistent with the reported results for a series of paired breast primary carcinoma and lymph-node metastases [57]. Although the number of clinical samples examined here is insufficient to allow for statistical analysis of whether the relative expression of this miRNA may predict different clinical outcomes in patients, the observation that its expression is severely reduced in metastatic lesions compared with primary localized tumors supports the oncosuppressive role of miR-214-3p in EWS.

In in vitro experiments, we confirmed that whenever we deprived EWS cells of EWS-FLI1 or CD99, either stably, transiently or by using a specific antibody against CD99, the expression of miR-214-3p increased. Its re-expression significantly suppressed cell proliferation and migration. Mechanistically, increased levels of miR-214-3p led to decreased expression of HMGA1 protein, a chromatin architectural protein that was demonstrated to be a direct target of miR-214-3p in other tumors [47,48]. HMGA1 does not have transcriptional activity per se but can modify chromatin structure by interacting with the transcriptional machinery and regulating the expression of many genes [58]. HMGA proteins are expressed at low levels in differentiated adult cells but at very high levels in embryonic cells and cancer [35]. In addition, in many carcinomas, such as breast, colon, lung, and ovary cancers, the expression level of HMGA1 was found to inversely correlate with the clinical prognosis [59,60,61]. Several studies have indeed demonstrated that HMGA1 expression is associated with tumor growth and metastasis by regulating several signaling pathways, such as those involving p53, STAT3, Cyclin D1, and CyclinE1 [47,62,63,64,65]. Additionally, HMGA1 confers resistance to several chemotherapeutic agents [66,67,68]. In EWS, when HMGA1 was repressed by miR-214-3p, cells showed decreased cell growth and migration, indicating that the suppression of miR-214-3p may allow HMGA1 to play a role in sustaining tumor aggressiveness.

## 5. Conclusions

Overall, our results support a model in which the two hallmarks of EWS (EWS-FLI and CD99) negatively regulate the expression of the miR-214-3p. Its restoration leads to inhibition of HMGA1 expression, leading to the inhibition of cell growth and migration. The inverse relationship between the expression of miR-214-3p and tumor aggressiveness was also demonstrated in clinical settings: metastatic lesions barely expressed this miRNA while localized primary tumors exhibited high levels. This study presents evidence that miR-214-3p acts as a potential tumor suppressor in EWS.

## Data Availability

All the data generated or analyzed during this study are included in this manuscript and its Supplementary Materials.

## Supplementary Materials

The following supporting information can be downloaded at: https://www.mdpi.com/article/10.3390/cancers14071762/s1. Table S1: List of miRNAs identified in the three databases. Table S2: List of cell lines used in this study. Figure S1: Protein expression analysis of some validated targets of miR-214-3p. Figure S2: Anchorage-independent growth of EWS cell lines transiently or stably transfected with pre- miR-214-3p. (A) Anchorage-independent growth of TC-71 (left) and IOR-CAR (right) EWS cell lines transfected transiently with pre-miR-214-3p mimic. Each column represents the mean ± SE of three independent experiments (* *p* < 0.05; ** *p* < 0.01, one way ANOVA test). (B) Anchorage-independent growth of SK-N-MC after stable transfection. Each column represents the mean ± SE of three independents experiments (*** *p* < 0.001, one way ANOVA test). Figure S3: HMGA1 expression analysis by q-RT-PCR after treatment of TC-71 cells with the anti-CD99 antibody mAb 0662 (3 µg/mL). Data are shown as 2^−ΔΔCT^ using TC-71 cells for normalization and *GAPDH* as endogenous control. The data are shown as the mean ± SE of three independent biological experiments performed in duplicate (* *p* < 0.05, ** *p* < 0.01; *** *p* < 0.001 one-way ANOVA).
