# Supplementary material for: miR-214-3p Is Commonly Downregulated by EWS-FLI1 and by CD99 and Its Restoration Limits Ewing Sarcoma Aggressiveness
Source: Cancers (Basel). 2022 Mar 30;14(7):1762. doi: 10.3390/cancers14071762 (PMC8997046; doi:10.3390/cancers14071762)
Supplement: Supplementary file 1 [file cancers-14-01762-s001.zip › Supplemental Table S2.pdf]

Supplemental TableS2: List of cell lines used in this study

Cell lines	Origin	Modification	References
**HS128, HS196, HS244, HS249, HS128**	Human bone-marrow derived mesenchymal stem cells		Amaral A.T. et al PLoS One 2014, 9, e85814.
**LAP-35**	Ewing sarcoma		Bagnara et al. Int. J Cell Cloning 1990 Nov;8(6):409-24
**A673**	Ewing sarcoma		CLS Cat#300454/p491_A-673
**IOR-BRZ**	Ewing sarcoma		Guerzoni C. et al. Clin Cancer Res 2015, 21, 146-156.
**IOR-CAR***	Ewing sarcoma		Guerzoni C. et al. Clin Cancer Res 2015, 21, 146-156.
**RD-ES**	Ewing sarcoma		CLS Cat#300410/p763_RD-ES
**SK-ES-1**	Ewing sarcoma		CLS Cat# 300435/p738_SK-ES-1
**TC-71 ***	Ewing sarcoma		Provided by T.J Triche (Children's Hospital, Los Angeles).
**SK-N-MC**	Ewing sarcoma		ATCC Cat# CRL-2270
**PDX-EW#5-C***	PDX Ewing sarcoma		Nanni et al. Sci Rep 2019, 9,12174
**PDX-EW#2-C***	PDX Ewing sarcoma		Nanni et al. Sci Rep 2019, 9,12174
**TC-71-CD99shRNA**	Ewing sarcoma	shRNA plasmid (pSilencer 2.1-U6 Neo vector; Ambion) (5'-GATCCGGCTGGCCATTATTAAGTCTTCAAGAGAGACTTAATAATGGCCAGCCTTTTTGGAAA-3') using the calcium phosphate transfection method.	Rocchi A et al. J Clin Invest 2010, 120, 668-680.
**A673/TR/shEF**	Ewing sarcoma	shRNA plasimid (pENTR-BLOCK-iT; Invitrogen) (shEF forward, 5'-CACCGCAGCAGAACCCTTCTTATGACGAATCATAAGAAGGGTTCTGCTGC-3'; shEF reverse, 5' AAAAGCAGCAGAACCCTTCTTATGATTCGTCATAAGAAGGGTTCTGCTGC-3 using lentivirus method	Carrillo J. et al. Clin Cancer Res 2007, 13, 2429-2440.
**A673p6TR/pTERshCD99**	Ewing sarcoma	pcDNA/6TR (Thermo Fisher Scientific) encoding the reverse tetracycline-responsive transcriptional activator and the plasmid pTER vector, and engineered to express CD99 shRNA Transfection was performed using TransIT-X2 (MIR 6000, Mirus)	Balestra T et al. Mol Cancer Ther 2022 21(1):58-69).
**SK-N-MC EV**<br>**SK-N-MC miR-214-3p#12**<br>**SK-N-MC miR-214-3p#34**	Ewing sarcoma	pcDNA3.1/V5-His-Topo-expression vector (Invitrogen),miR-214 5'CACCTTTCTCCCTTTCCCCTTACTTACTCTCC-3' and 5' TGCCTTTCCCCAGTGCCTCTTTCTC-3'. Transfection was performed usincLipofectamine 2000 (Thermofisher)	Provided by H. Kovar (St. Anna Children's Cancer Research Institute, Vienna).

*TC-71, IOR-CAR and PDX-EW#2-C cells were transiently transfected with pre-miR-214-3p mimic, and PDX-EW#5-C cells were transfected with antagomiR-214-3p or nonspecific control miRNAs (SCR) (30 nM) (assays #AM17100 and #AM171000; Ambion) and siHMGA1(20nM) (M-004597-02-0020 GE Healthcare Dharmacon)or si-GENOME_nontargeting (D-001206-13-05, GE Healthcare Dharmacon) using a TransIT-X2 Dynamic Delivery System (Mir6000, Mirus).
