# Supplementary figures and images for: miR-214-3p Is Commonly Downregulated by EWS-FLI1 and by CD99 and Its Restoration Limits Ewing Sarcoma Aggressiveness
Source: Cancers (Basel). 2022 Mar 30;14(7):1762. doi: 10.3390/cancers14071762 (PMC8997046; doi:10.3390/cancers14071762)

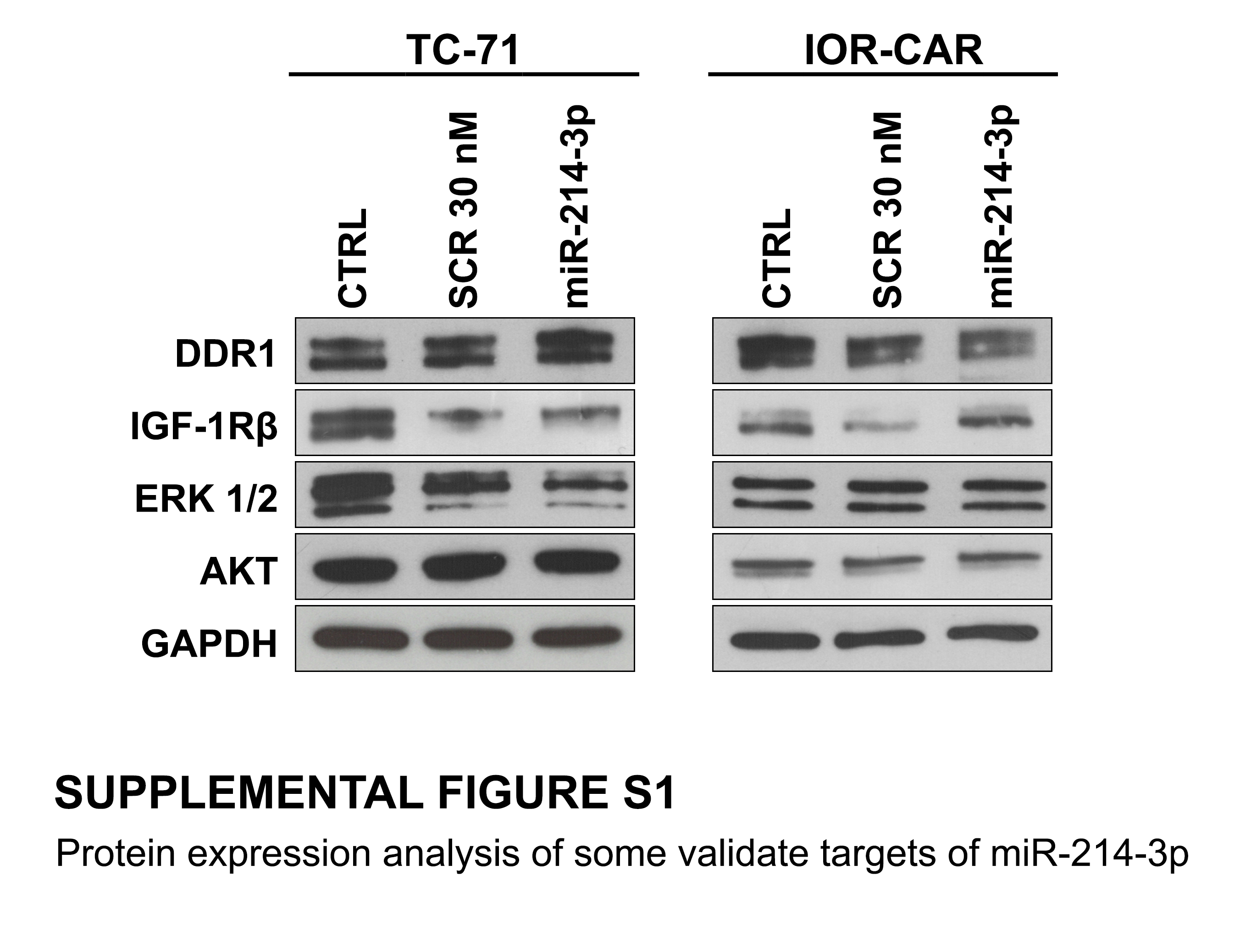

Supplement: Supplementary file 1 [file cancers-14-01762-s001.zip › SUPPLEMENTAL FIGURE S1.tif]

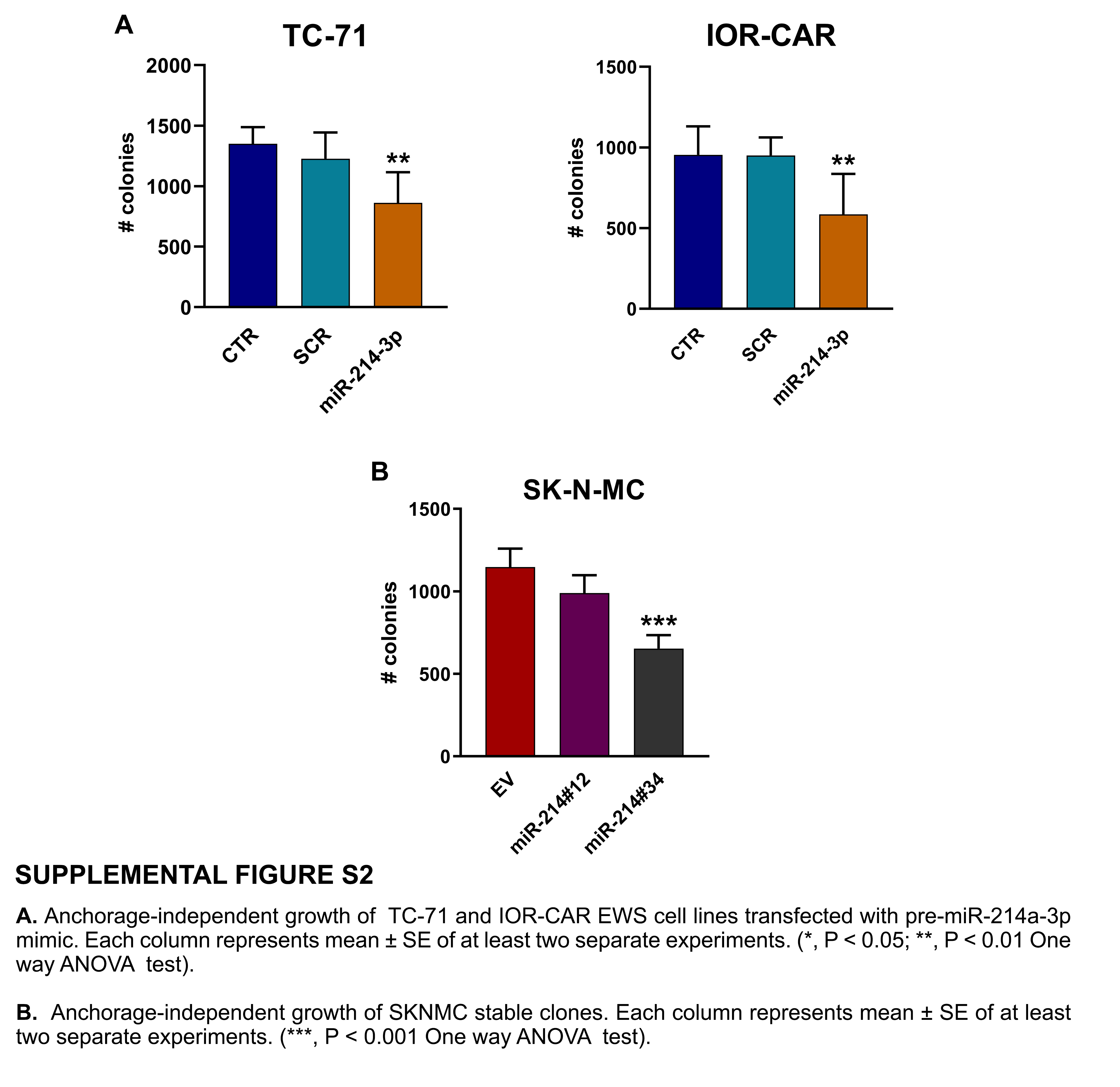

Supplement: Supplementary file 1 [file cancers-14-01762-s001.zip › SUPPLEMENTAL FIGURE S2.tif]

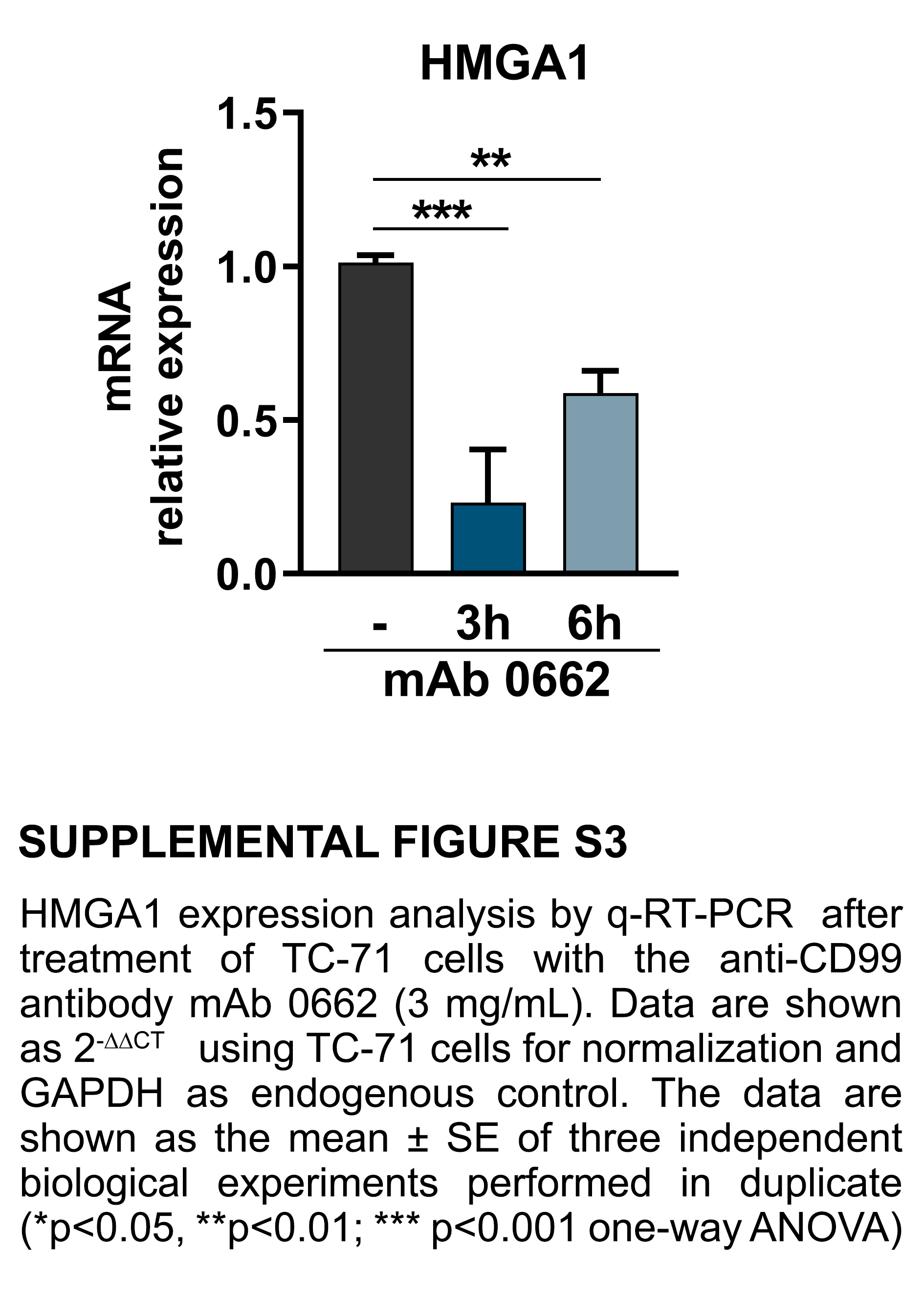

Supplement: Supplementary file 1 [file cancers-14-01762-s001.zip › SUPPLEMENTAL FIGURE S3.tif]
